# Converting Endangered Species Categories to Probabilities of Extinction for Phylogenetic Conservation Prioritization

**DOI:** 10.1371/journal.pone.0003700

**Published:** 2008-11-11

**Authors:** Arne Ø. Mooers, Daniel P. Faith, Wayne P. Maddison

**Affiliations:** 1 Institute for Advanced Study, Berlin, Germany; 2 Biological Sciences, Simon Fraser University, Burnaby, British Columbia, Canada; 3 The Australian Museum, Sydney, Australia; 4 Biodiversity Research Centre, University of British Columbia, Vancouver, British Columbia, Canada; University of Kent, United Kingdom

## Abstract

**Background:**

Categories of imperilment like the global IUCN Red List have been transformed to probabilities of extinction and used to rank species by the amount of imperiled evolutionary history they represent (e.g. by the Edge of Existence programme). We investigate the stability of such lists when ranks are converted to probabilities of extinction under different scenarios.

**Methodology and Principal Findings:**

Using a simple example and computer simulation, we show that preserving the categories when converting such list designations to probabilities of extinction does not guarantee the stability of the resulting lists.

**Significance:**

Care must be taken when choosing a suitable transformation, especially if conservation dollars are allocated to species in a ranked fashion. We advocate routine sensitivity analyses.

## Introduction

The World Conservation Union (www.iucn.org) is the largest and most influential conservation network in the world. One of its most influential products is the ‘Red List’, a quantitative categorization of the global level of imperilment for individual species (see, e.g., www.iucn.org/themes/ssc/redlist2007/index_redlist2007.htm). Using multifaceted criteria [1], [2], the IUCN designates species as being in one of a number of conservation categories, ranging from ‘Least Concern’ to ‘Extinct in the Wild.’ Though controversial [3], conservation organizations and different levels of government use both the criteria and the lists when planning conservation interventions [3], [4]. For instance, both IUCN categories and global assessments are used by the Committee on the Status of Endangered Wildlife in Canada when ranking species under the Canadian Species at Risk Act (see www.cosewic.gc.ca).

In addition, researchers have successfully used this list to explore geographical (e.g. [5]) and biological (e.g. [6]) correlates of extinction risk. For such comparative studies, the categories are treated as ranks such that species of same rank are considered equivalent.. Because these studies generally rely on non-parametric approaches, no assumptions are needed about the change in extinction probability between ranks, though their results might be interpreted as if differences between ranks (e.g. from LC to NT and from EN to CR) are assumed equivalent.

There are, however, contexts in which we need more than species ranks. Whenever the IUCN species ranks are blended with other criteria such as cost of recovery and probability of success to generate a quantification of conservation priority, the ranks need to be assigned numerical values that represent interpretable measures such as extinction probability. Such studies are likely to become more common. IUCN categories are now more generally applicable, given that museum collections and related data provide a way to assess one of the criteria, geographic range size, for many different species [7].

Another important context, of interest in this study, is the integration of IUCN categories with phylogenetic trees. The ranks for different species can be combined to determine the probability of loss of deeper evolutionary history (shared branches on the phylogentic tree) only when the ranks are interpreted as probabilities of extinction. For example, recent work has used extinction risks to project expected losses to a phylogenetic tree [8], [9] and these projected losses have been be combined with other quantified considerations to help choose subsets of species that maximize total phylogenetic variation [10], [11]. The impetus for this work is the recognition that the loss of some species represents a disproportionate loss of evolutionary history. Recently in this journal, Isaac et al. [12] presented the EDGE (Evolutionary Distinct, Globally Endangered) metric to direct practical global conservation action (www.edgeofexistence.org). EDGE combines a measure of a species' isolation on a phylogenetic tree with a measure of a species current imperilment. Technically, it is a logarithmic transformation of the product of a species' evolutionary distinctiveness and the probability it will go extinct [13]. An alternative measure, the ‘heightened’ EDGE or HEDGE score [14] includes the probabilities of extinction of other species in the tree, and can be formulated as the expected gain one can make in evolutionary history preserved by protecting a species (see also [15]). In both cases, explicit probabilities of extinction are required, and Redding and Mooers [13] and Isaac et al. [12] have suggested ways to transform the Red List categories to prob(extinction). The Red List is currently the only basis we know of for consistent, broadly-available estimates of extinction risk, and indeed was originally formulated to be consistent with (at least) notional probabilities of extinction [1], [2], [16].

We support the quantification of conservation importance. The above metrics (EDGE and HEDGE) clearly define species priority ranks for a fixed tree and fixed probabilities of extinction (both of which can change with new information). However, in this short note we highlight how the choice of transformation of Red List categories to different probabilities of extinction (see, e.g. Table 1) can affect the resulting species rankings for both the above metrics, sometimes dramatically. We urge practitioners to use great care when performing any transformation of ranks. The underlying reason for this sensitivity to both metrics is the same: ranks are not enough.

**Table 1:** Parameters used to test sensitivity of ranks-to-extinction probability transformations.

			Extinction Probabilities			
IUCN Category	% tips^1^	Isaac^2^	IUCN100^3^	IUCN50^4^	IUCN500^4^	Pessimistic
Least Concern	76	0.025	0.0001	0.00005	0.0005	0.2
Near Threatened	9	0.05	0.01	0.004	0.02	0.4
Vulnerable	9	0.1	0.1	0.05	0.39	0.8
Endangered	4	0.2	0.667	0.42	0.996	0.9
Critically Endangered	2	0.4	0.999	0.97	1	0.99

1 Mean proportions of species in each IUCN category across birds and mammals.

2 Inferred from [12], using the IUCN designation of Prob(ext)_VU_ = 0.1 in 100 years.

3 Projected Prob(extinction) at 100 years using IUCN designations (IUCN, 2001): Prob(ext)_CR_ = 0.5 in 10 years; Prob(ext)_EN_ = 0.2 in 20 years; Prob(ext)_vu_ = 0.1 in 100 years. LC and NT categories interpolated; see methods.

4 IUCN designations projected to 50 or 500 years.

The transformations of categories to probabilities of extinction requires two pieces of information. The first is the relative difference among categories–i.e. does the movement among categories reflect a constant change in prob(extinction) (e.g. by factor 2, as presented by Isaac et al. [12]), or is the relationship nonlinear (e.g. as presented by Redding and Mooers [13] or the IUCN itself)? Second, if we consider extinction as a Poisson process, then the categories could be interpreted as instantaneous rates (rather than probabilities), and then the time scale for conservation can have drastic effects on the absolute and on the relative p(extinction) [17]. We explore both issues here.

**Figure 1:**
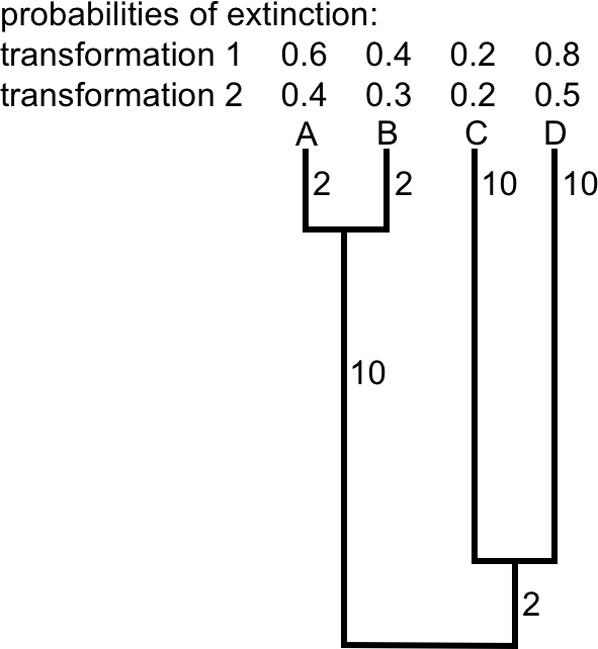
Changing EDGE and HEDGE scores. EDGE_i_ = ln(ED_i_*Prob(extinction)_i_), where ED_i_ is the sum of edge lengths from the root of the tree to i, each edge length divided by the size of the clade the edge subtends. In figure 1, ED_A_ = ED_B_ = 10/2+2/1 = 7, and ED_C_ = ED_D_ = 2/2+10/1 = 11. Each species is in a different category of extinction risk (in the rank order of imperilment D,A,B,C ), and these categories have been transformed to prob(extinction) under two scenarios: in the first (power) transformation of extinction categories (Transformation 1), the rank order under EDGE is D (2.2), A (1.4), B(1.0) and C(0.8). With the second (linear) transformation (Transformation 2), C and B switch ranks: D(1.7), A(1.0), C(0.8), and B(0.7). HEDGE_i_ is also a product of two terms: the expected phylogenetic contribution of a species *i* given prob(extinction) of all other species, and prob(extinction)_i_. However, HEDGE can also be formulated as the change in the total expected PD [28] from the status quo to the expected PD if one sets the prob(extinction)_i_ = 0, ie. if species *i* is preserved [15]. The expected PD of a tree can be calculated as the sum of edge lengths, each weighted by its probability of persistence [8]. For example, the expected PD under transformation 1 is 2*0.4+2*0.6+10*(1−0.6*0.4)+0.2*10+0.8*10+(1−0.2*0.8)*2 = 21.3; if we save C, it becomes 2*0.6+2*0.4+10*(1−0.6*0.4)+1*10+0.2*10+(1−0*0.8)*2 = 23.6, and HEDGE_C_ = 23.6−21.3 = 2.3. The ranked HEDGE list for the species in Figure 1 under transformation 1 is D(8.32), A(3.6), B(3.2), C(2.3). Under transformation 2, the ranked list becomes quite different, with C moving from last to second place: D(5.2), C(2.2), A(2.0), B(1.8).

Figure 1 presents a four-species tree on which EDGE and HEDGE return different static species rankings under two simple transformations of fictional Red List categories (see Figure 1 legend). For these small trees, both EDGE and HEDGE measures can be calculated readily by hand. Under the first transformation, the rank order for EDGE is DABC, while, under the second, it is DACB; the ranked HEDGE list for the species in Figure 1 under transformation 1 is also DABC; under transformation 2, it becomes DCAB.

This contrived example, however, might not be typical. We therefore used simulated trees and current IUCN data for mammals and birds to ask how strongly both EDGE and HEDGE rankings are affected by four transformations of the five most common Red List categories (LC, NT, VU, EN, and CR) to quantitative values for predicted prob(extinction) (see Table 1). The first transformation draws on Isaac et al., scaled to a common time scale of 100 years; the second transformation follows the IUCN designations themselves [18] with interpolation, and draws a stronger contrast among categories; the third and fourth are simple extensions of the IUCN designations but scaled to 50 and 500 years. Finally, we include an arbitrary “pessimistic” transformation that designates a sizable prob(extinction) = 0.2 even for the ‘least concern’ species for comparison. We report summary statistics (see methods) for three comparisons: the published Isaac et al. transformation vs. the published IUCN transformation (both scaled to a 100 year window); the Isaac et al. transformation vs. the arbitrary pessimistic transformation; and two transformations that differ solely on the time-window used: IUCN50 vs. IUCN500.

## Results

Table 2 presents the summary statistics of our simulations (full simulation results are available from the first author upon request). When measured using the summed differences, the HEDGE metric is more sensitive to transformations in general. In addition, although all three transformation comparisons give similar results when one considers the percent of trees that differ, the summed differences for the comparison of the two ‘standard’ transformations (Isaac vs. IUCN, measured on the same 100-year interval) differ much more than do transformations that only involve time-window shifts.

**Table 2:** Results of sensitivity of ranks-to-extinction probability transformations.

		Top 5 species		Top 20 species	
Metric	Transformation Comparison	% trees that differ^1^	Sum of differences^2^	% trees that differ^1^	Sum of differences^2^
EDGE	Isaac vs. IUCN100	4.15 (0.08)	9.7 (0.7)	17.0 (0.1)	114 (4)
	Isaac vs. Pessimistic	3.11 (0.09)	27.8 (1.1)	19.8 (0.04)	92.6 (3)
	IUCN50 vs. IUCN500	4.0 (0.07)	15.8 (0.6)	20 (0)	26.8 (1)
HEDGE	Isaac vs. IUCN100	3.51 (0.09)	28 (1.8)	11.8 (0.2)	417 (10)
	Isaac vs. Pessimistic	3.84 (0.08)	23.7 (1.9)	16.9 (0.1)	181 (7)
	IUCN50 vs. IUCN500	3.83 (0.08)	16.1 (0.7)	19.8 (0.06)	56 (2)

Entries are the average (standard error) across 100 100-tip birth-death trees.

1 Mean percent of the 100 trees that differ in the identity of the top 5 (or 20) ranked species under contrasting transformations. Standard errors in brackets.

2 The mean across 100 trees of the sum of the differences in the ranks between two transformations. Standard errors in brackets.

## Discussion

In our heuristic example in which both the tree and the conservation designations remained constant, species D consistently ranked first–it is a relatively unique species with the highest risk of extinction; the fairly redundant and mildly imperiled species A and B always rank in the same relative order, while C, a distinctive but safe species related to another distinct but imperiled species is the most volatile, finishing ahead, between or behind A and B depending on the transformation and metric used. Under HEDGE, C moves between transformations from least important to second-most important species.

EDGE and HEDGE are superficially different metrics of species value. However both can be written as a product with two terms. The first is some measure of how much non-redundant phylogenetic information species *i* represents presently (ED) or will represent in some defined future (HED). The second term might be termed an ‘urgency score,’ ie the current prob(extinction) of species *i*. As such, it is clear that as probabilities of extinction change, so too will species values. Given the uncertainty associated with prob(extinction)–both in assigning current probabilities and in projecting those into the future, we had hoped the tree would dominate calculations such that ranks would have been robust against transformations. However, one can get differences while retaining rank information (e.g. retaining the IUCN designations). Transformations differing only by time window (i.e. a 50 versus a 500-year perspective) lead to more similar outcomes than do other transformations. However, because it is hard to avoid using extinction probabilities in a quantitative conservation framework, both for assessing urgency and for estimating the importance a species will have in representing future phylogenetic diversity, the general pattern is sobering.

Our results suggest that HEDGE is somewhat more sensitive to different transformations. This makes sense, since the prob(extinction) values are used both to calculate the expected future tree lengths and the focal species' urgency score. EDGE is therefore a somewhat more ‘robust’ metric. However, we do not place too much emphasis on this difference in performance: Faith [15] has argued clearly that HEDGE belongs in a “probabilistic PD ” framework that may better achieve the goal of conserving future variety. Further, he argued that these measures logically may return varying priorities when integrated into “phylogenetic risk analyses” that reflect varying degrees of risk-aversion to worst-case losses of evolutionary history. Robustness is not necessarily an asset if volatility is due to considerations that really matter.

This study did also not look at the sensitivity of these ranking metrics to incorrect or imprecise ED scores for a species. This is not because we believe ED scores are easier to estimate. First, (H)EDGE scores are ‘expected loss’ scores, that is an evolutionary value attached to a species multiplied by the probability that it the will be lost to extinction. The redundancy in phylogenetic trees means that ED scores may often vary less (and have less of an effect on the metrics) than prob(extinction), though this will vary greatly among clades. The standard IUCN scoring we used spans 4 orders of magnitude, while Madagascar primate ED scores span <1, all primates <2, and all 4500 mammal species, ∼4 orders of magnitude.

We also know from previous work that most evolutionary distinctiveness metrics, including the one we use here, are heavily weighted by the branches nearest the tips [19]. This means that mistakes deeper in the tree will not generally have large effects on ED scores. It also means that what is designated as a ‘tip’ worthy of independent conservation attention is critical: splitting a species into two decreases the evolutionary distinctiveness of each quite dramatically [12].

Finally, we suggest that work continue in the area of representing phylogenetic redundancy using a measure more refined than elapsed time (see [20]). Representing ecological or morphological or genetic divergence on an additive tree would produce a broader range of ED values, compensating for the weight currently placed on prob(extinction).

As limited resources are targeted to conservation efforts, economic models to assign priorities to these efforts will come to have increasing importance. The quantitative interpretability of the models' components, such as IUCN ranks, is vital. There are alternative methods for estimating prob(extinction) for species [21]. We therefore suggest that quantitative conservation frameworks, and phylogenetic conservation approaches more specifically, consider more closely how prob(extinction) values are derived. We do not advocate any particular set of transformations from IUCN rankings, but we echo suggestions [22]–[24] that quantitative frameworks take uncertainties in those prob(extinction) values into account when designing specific conservation strategies (see also [25]). Uncertainties arise both from the point estimates of prob(extinction) and from the time frame over which prob(extinction) values are extended. For example, it seems reasonable to consider shorter time horizons for species with more dynamic demographics. Sensitivity analyses should be done routinely for any and all metrics that are used to identify or rank species for conservation attention: here, prob(extinction) values could be drawn from reasonable distributions both within and across IUCN (or other) ranks and species that consistently rank highly be given higher priority. This would be easy to do, and it may be possible to present the results to the public as a conservative approach based on the precautionary principle.

## Materials and Methods

We assigned the five main IUCN risk levels to the tips of 100 100-species birth death trees (b = 0.1, d = 0.06), in the same proportion per level as mean for the birds and mammals of the world [12], [13]; see our Table 1. We then converted each species' level to a probability of extinction under each of five transformations: one where each increase in level corresponds to a doubling of extinction risk [12] three transformations corresponding to the official IUCN designations, but scaled to 50, 100, and 500 year windows, and a pessimistic transformation of our choosing. The IUCN has not designated prob(extinction) for the two lowest categories, and these had to be interpolated. Partly in order to produce contrasting scales, we set prob(extinction) for the ‘least concern’ species to 0.01% [13], equivalent to assuming that at most 1 of the 7600 bird species in this category would go extinct over the next 100 years; the Near Threatened category was given a prob(extinction) 100 times this, in accord with the interpolation used in [13].

For each tree and assignment, we calculated the EDGE and HEDGE scores using the Tuatara module [26] of the Mesquite package [27]. We asked how often the top ranked species differed as one moved between transformations. When the ranks differed between transformations, we also recorded the degree of this difference by taking the sum of the differences in ranks. For example, if the top five species under the Isaac transformation {1,2,3,4,5} are ranked {1,5,3,10,2} under the IUCN100 transformation, this contributes 12 (0+3+0+6+3) to the sum, and if the top five species under the IUCN100 transformation {1,2,3,4,5} are ranked {1,5,3,8,2} under the Isaac et al. transformation, this contributes 10 {0+3+0+4+3}, giving a summed difference score of 22. We considered four measures of sensitivity to transformation. For the top five- and for the top 20-ranked species under a transformation, we recorded the proportion of the simulated trees that showed any difference, and also the average across trees of the sum of these differences in ranks.
